# Maternal Mortality in Nepal: Identifying High-Risk Groups Through Census Data

**DOI:** 10.1007/s10900-026-01562-1

**Published:** 2026-03-06

**Authors:** Monna Kurvinen, Sharad Sharma, Keshab Deuba

**Affiliations:** 1 Public Health and Environment Research Center (PERC), Lalitpur, Nepal; 2 National Statistics Office, Office of the Prime Minister and Council of Ministers, Government of Nepal, Kathmandu, Nepal; 3 Centre for International Health (CIH), Department of Global Public Health and Primary Care, University of Bergen, Bergen, Norway; 4 Department of Global Public Health, Karolinska Institutet, Stockholm, Sweden

**Keywords:** Maternal mortality, Maternal death, Low- and middle-income countries, Nepal

## Abstract

Nearly all maternal deaths are preventable, yet Nepal’s maternal mortality ratio remains high, with social and geographic disparities. Beyond direct obstetric causes, factors such as education, income, and place of residence influence maternal mortality risk, but disaggregated national data remain limited. This study examined the social determinants and timing of maternal deaths in Nepal to identify high-risk groups and inform targeted interventions. A cross-sectional descriptive study was conducted using microdata from the 2021 National Population and Housing Census, applying a stratified, probability-based sampling design to ensure national representativeness. Deaths among women aged 15–49 were identified through household reports. Data were collected on sociodemographic characteristics, household wealth, ethnicity, and place of residence. Deaths were classified by cause, and maternal deaths were further classified by timing, including during pregnancy, childbirth, or within six weeks postpartum. Descriptive statistics were calculated using sampling weights. Of 1,386 deaths among women aged 15 to 49 years, 54 (3.9%) were maternal deaths, with 74.6% occurring within six weeks postpartum. Non-communicable diseases were the leading cause of death overall (49.9%). Maternal deaths were most frequent in Lumbini province, peri-urban and rural areas, and the Tarai region. Socioeconomic disparities were evident, with nearly half of maternal deaths occurring among women from poor households. Most women who died from maternal causes were aged 20–34 years. Maternal mortality in Nepal reflects social and geographic inequalities, with higher risk among poorer women, those in rural and peri-urban areas, and specific provinces, particularly during the postpartum period. Strengthening antenatal care, skilled birth attendance, and equitable, context-specific interventions is essential to reduce preventable maternal deaths.

## Introduction

Nearly all maternal deaths are preventable through evidence-based interventions that are accessible, affordable, and delivered via quality health services [1]. Nonetheless, maternal mortality remains a critical global public health challenge, with the Sustainable Development Goal aiming to reduce the global maternal mortality ratio (MMR) to less than 70 deaths per 100,000 live births by 2030 [2]. Despite notable progress, Nepal continues to experience a high MMR of 151 per 100,000 live births, with provincial rates ranging from 98 to 207 [3], lagging behind neighbouring countries such as Bhutan (47), China (16), India (80), and Bangladesh (115) [1]. These figures indicate that Nepal is in an intermediate stage of the maternal mortality transition [4]. Although substantial advances have been made in social development, health system coverage, and skilled birth attendance, significant gaps remain in the quality of care [4]. The health system continues to struggle in managing obstetric emergencies and addressing pre-existing risk factors and external exposures associated with maternal death and morbidity [4].

While common causes of maternal death, such as haemorrhage, pre-eclampsia, and unsafe abortion, remain critical, they do not occur in isolation [4]. These are downstream results shaped by a range of broader social, economic, environmental, and structural factors [4]. Achieving further reductions in maternal mortality, therefore, requires greater attention to these underlying determinants, which influence women’s access to and utilization of sexual and reproductive health services [1]. Factors such as education, ethnicity, income, and geographic location have been shown to strongly predict maternal mortality and morbidity [1]. However, progress is limited by insufficient data on these determinants and exposures, highlighting the need for more disaggregated information by socioeconomic status, rural/urban residence, and ethnicity to detect inequalities and inform targeted interventions [4, 5].

The 2021 Nepal Maternal Mortality Report [3] identified non-obstetric complications, so-called indirect maternal deaths, as the leading cause of maternal mortality (32%), followed by haemorrhage (25%). Notably, a majority of maternal deaths occurred in health facilities (57%), with another 26% in private residences, indicating that while access to birthing units is relatively high, the quality of care provided remains suboptimal.

There is a pressing need for more comprehensive data on the determinants, exposures, and risk factors influencing maternal health to better detect inequalities. Future research is also needed to identify which determinants have the greatest impact across provinces, ecological regions, and population groups, and to understand how progress through different stages of the maternal mortality transition can be accelerated in the Nepali context [4]. Addressing inequities explicitly is essential, and structural discrimination based on gender, ethnicity, or other social factors must be recognized and systematically addressed in policy and programme design [4].

This study aims to examine the social determinants and timing of maternal deaths in Nepal to identify high-risk groups and priority areas for prevention and quality-of-care improvement.

## Methodology

### Study Design and Setting

This cross-sectional descriptive study utilized secondary data from the 2021 National Population and Housing Census (NPHC) [6]. The census was conducted under Nepal’s federal governance structure, which comprises seven provinces, 77 districts, 753 municipalities, and 6,743 wards [7].

### Census and Microdata Sample

The NPHC is an integral component of Nepal’s national statistical system. It provides the benchmark for population counts at both national and sub-national levels, conducted every ten years. For small geographic areas or specific sub-populations, the census may serve as the only source of reliable information on social, demographic, and economic characteristics. This study utilized microdata from the 2021 NPHC, enabling in-depth analysis of population and housing dynamics in Nepal.

### Sampling Strategy

The microdata sample was designed to produce domain-disaggregated, representative datasets while maintaining precision and efficiency. A probability-based stratified sampling approach was employed to generate robust estimates up to the municipal level. However, mortality estimates from the sample are not recommended below the provincial level, as the number of sampled cases is too small to yield reliable results. The minimum required sample size for valid statistical estimates was determined using Cochran’s formula, widely applied in survey sampling to estimate proportions with specified levels of precision and confidence.

### Study Population and Sample Size

The census recorded a total of 622 maternal deaths, and this analysis is based on a selected microdata sample of 62 maternal deaths, corresponding to 54 deaths after applying sampling weights. Overall, 1,429 deaths among Nepali women aged 15–49 years were identified, representing 1,386 deaths after weighting. The use of weights ensures that the estimates accurately reflect the true population parameters, making the findings generalizable at both the national and provincial levels.

### Data Collection

The NPHC 2021 was a complete national census, and the main questionnaire, which forms the basis of this study, was administered to all usual resident households in Nepal, covering a total of 6,666,937 households [6]. Data were collected across four thematic areas: (1) household information, (2) individual information, (3) information on absentees, and (4) information on deceased persons within a 12-month reference period [6]. These areas captured detailed information on household characteristics, facilities and amenities, and socio-demographic and economic factors, including education, migration, economic activity, occupation, industry, women’s empowerment, fertility, and mortality [6]. Data collection was conducted from November 11 to November 25, 2021 [6].

### Variables

#### Sociodemographic Characteristics

Household members provided information on the deceased women’s age, as well as their family members’ province, type of place of residence (urban, peri-urban, or rural), ecological belt (mountain, hill, or Tarai, characterized by plains and an open border with India), household wealth, ethnicity, and religion, which were assumed to reflect those of the deceased women. Frequencies and percentages were used to describe variations in these sociodemographic characteristics.

Age was categorized into four groups: 16–19, 20–24, 25–34, and 35 years or older. Household wealth was assessed based on their ownership of consumer goods and housing characteristics, such as televisions, water source, and toilet facilities. Each household was assigned a wealth score and ranked accordingly. Finally, the population was divided into five equal groups, each representing 20% of the total, to create national wealth quintiles. The first two wealth quintiles were combined into the “poor” category, the third and fourth wealth quintiles are grouped as “middle”, and the fifth wealth quintile is classified as “rich”.

### Outcomes of Interest

The cause of death was reported for all deceased women aged 15–49 years. Maternal deaths within the past 12 months were identified through verbal autopsy interviews conducted with family members, who were specifically asked about the condition of deceased women at the time of death. Each verbal autopsy was independently reviewed by two specialists (gynaecologists/obstetricians), who assigned the cause of death according to the International Classification of Diseases for Maternal Mortality (ICD-MM) guidelines. In cases of disagreement, a third reviewer independently assessed the case and assigned a cause of death. The final cause of death was determined by majority rule, with the cause assigned by at least two reviewers considered final. Maternal death was defined as a death occurring during pregnancy, childbirth, or within six weeks postpartum. The timing of maternal death, categorized as occurring during pregnancy, at childbirth, or within six weeks postpartum, was based on family members' reports.

### Statistical Analysis

Descriptive statistics were used to summarize the causes of death of women aged 15–49, the key sociodemographic characteristics of women who died from maternal causes, and the timing of maternal deaths in Nepal. Frequencies, percentages, and means were calculated, with 95% confidence intervals reported. Missing values were excluded from the analyses. All statistical analyses were conducted using STATA/SE version 14 (StataCorp LLC, College Station, TX, USA). Sampling weights, normalized to the sample size, were applied throughout the analyses to ensure that sample estimates accurately reflect the population distribution.

### Ethical Considerations

This study used secondary, anonymized data from the 2021 NPHC. All data were provided without any individual-level identifiers accessible to the researchers. Because the analysis was based exclusively on existing census data and involved no direct contact with human participants, additional ethical approval was not required. The study followed established ethical principles for secondary data research, including the protection of privacy, confidentiality, and responsible use of population-level information. All analyses were undertaken to advance public health knowledge on maternal mortality and to support evidence-based policy and program development in Nepal.

## Results

In total, 1,386 deaths occurred among women aged 15–49 years, of which 3.9% (*n* = 54) were maternal deaths (Table 1). Non-communicable diseases accounted for the largest proportion of deaths (49.9%, *n* = 691), followed by communicable diseases (14.0%, *n* = 194). Among maternal deaths, the majority occurred within six weeks postpartum (74.6%, *n* = 40), while 22.8% (*n* = 12) occurred during pregnancy and only 2.6% (*n* = 1) during childbirth.

**Table 1:** Cause of death among deceased Nepali women aged 15–49, and the timing of maternal deaths

	*N*	%	CI
*Cause of death (n = 1,386)*			
Communicable disease	194	14.0	12.2–15.9
Non-communicable disease^a^	691	49.9	47.2–52.5
Road accident	36	2.6	1.9–3.6
Other accident	69	5.0	4.0–6.3.0.3
Maternal death	54	3.9	3.0–5.1.0.1
Crime/murder	23	1.6	1.1–2.5
Suicide	137	9.9	8.4–11.6
Others	159	11.5	9.9–13.3
Not reported	23	1.7	1.1–2.5
*Timing of maternal death (n = 54)*			
During pregnancy	12	22.8	13.3–36.3
During childbirth	1	2.6	0.5–13.1
Within six weeks of childbirth	40	74.6	60.9–84.7

CI – 95% confidence interval

^a^Deaths reported as due to natural causes were classified under non-communicable diseases

Table 2 summarizes the sociodemographic characteristics of women who died from maternal causes, taking sample weights into account. The mean age at death was 25.7 years, with most deceased women aged 20–24 (29.1%, *n* = 16) or 25–34 (44.4%, *n* = 24) years.

**Fig. 1:**
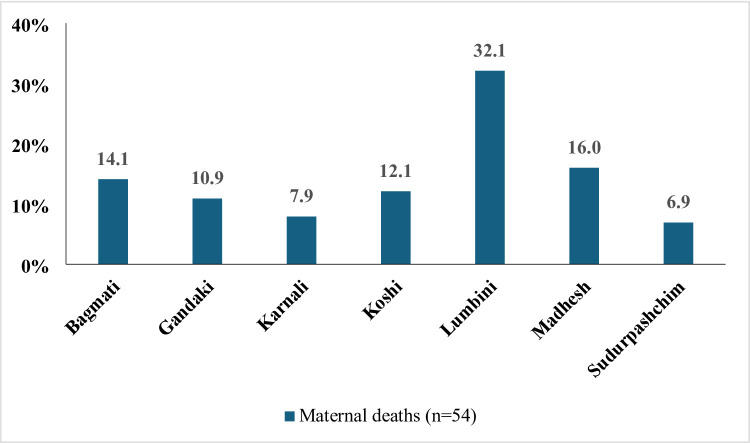
Maternal deaths (*n* = 54) by province

By province (Fig. 1), Lumbini accounted for the highest proportion of maternal deaths (32.1%, *n* = 17), while Sudurpashchim had the lowest (6.9%, *n* = 4). Maternal deaths were least prevalent in urban areas (17.1%, *n* = 9) and most frequent in peri-urban (42.8%, *n* = 23) and rural (40.1%, *n* = 22) settings. Regionally, over half of maternal deaths (52.7%, *n* = 29) occurred in the Tarai, followed by 43.2% (*n* = 23) in the Hill region.

**Fig. 2:**
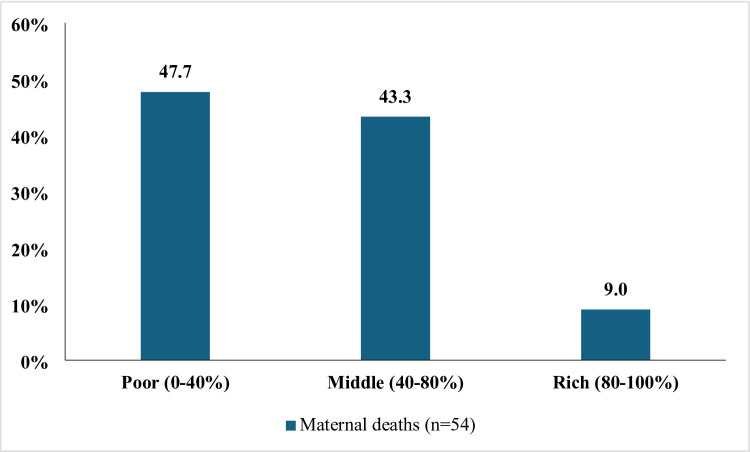
Maternal deaths (*n* = 54) by household wealth

Socioeconomic disparities were evident: only 9.0% (*n* = 5) of deceased women were from rich households, compared with 47.7% (*n* = 26) from poor and 43.4% (*n* = 23) from middle-income households (Fig. 2). The most represented ethnic group was the Mountain/Hill/Tarai Janajatis (30.7%, *n* = 17), while the least represented groups were Hill Castes (Brahmin/Kshetri) (12.0%, *n* = 6) and religious/linguistic groups (11.0%, *n* = 6). Regarding religion, most deceased women were Hindu (77.7%, *n* = 42).

**Table 2:** Sociodemographic characteristics of women aged 15–49 who died from maternal causes in Nepal

Variable	Maternal deaths (*n* = 54)		
	Mean (range)	%	CI
Age at death	25.7	N/A	24.1–27.2
	**N**		
*Age categories*			
16–19 years 20–24 years 25–34 years 35 + years	8 16 24 6	14.9 29.1 44.4 11.6	7.5–27.5 18.3–43.0 31.4–58.1 5.3–23.7
*Type of place of residence*			
Urban Peri-urban Rural	9 23 22	17.1 42.8 40.1	9.0–30.0 30.1–56.7 27.6–54.0
*Ecological belt*			
Mountain Hill Tarai	2 23 29	4.1 43.2 52.7	1.0–14.6.0.6 30.4–57.0 39.1–66.0
*Ethnicity*			
Hill Castes (Brahmin/Kshetri)	6	12.0	5.5–24.1
Madhesh/Tarai Caste	11	20.6	11.6–33.9
Mountain/Hill/Tarai Janajatis	17	30.7	19.6–44.6
Madhesh/Tarai/Hill Dalits	14	25.7	15.6–39.4
Religious/Linguistic	6	11.0	4.9–23.0
*Religion*			
Hindu	42	77.7	64.3–87.1
Buddhist	3	5.2	1.6–16.0
Islam	6	11.1	4.9–23.0
Kirat	3	6.0	2.0–17.0

## Discussion

This study examined maternal mortality and its social determinants in Nepal. The findings highlight several high-risk groups, including those from lower household wealth and residing in peri-urban or rural areas. Notable differences were also observed between provinces. Furthermore, the vast majority of maternal deaths occurred within the first six weeks postpartum, underscoring the critical importance of timely, high-quality, and accessible maternal care before, during, and after childbirth.

Non-communicable diseases accounted for the largest proportion of deaths among women aged 15–49 years, reflecting a well-documented global trend [8]. Rising levels of obesity are one of the key contributing factors, including in Nepal, and are closely linked to conditions such as diabetes and cardiovascular disease [9, 10]. Pre-existing non-communicable diseases are also associated with substantially increased risk of obstetric complications and maternal mortality [11, 12]. Strengthening population-based prevention and management strategies through early screening and lifestyle counselling, such as interventions promoting a healthy diet and regular physical activity, is essential. Communicable diseases were the second most common cause of death. Nepal is among the high-burden countries for tuberculosis, and the disease ranks among the top ten causes of mortality [13, 14]. Although maternal deaths constitute a relatively small proportion of all deaths among women of reproductive age, nearly all maternal deaths are preventable, underscoring the critical importance of timely, high-quality maternal and reproductive health services.

Maternal deaths were most frequent among women aged 25–34 years, likely reflecting higher exposure to pregnancy-related events due to higher fertility rates in this age group. However, previous studies indicate that both younger adolescents (< 18 years) and older women (> 35 years) face a disproportionately higher risk of maternal mortality [4]. These findings underscore the need for maternal health interventions that ensure timely access to high-quality antenatal, delivery, and postnatal care for women of all ages.

Differences between provinces were also evident. Lumbini had the highest share of maternal deaths, accounting for nearly one-third of all cases. The province’s disproportionately high maternal mortality highlights the need for targeted attention. According to the Nepal Maternal Mortality Report 2021, Lumbini’s maternal mortality ratio was an alarming 207 deaths per 100,000 live births, compared with the national average of 151 [3]. Although Lumbini has a relatively strong health infrastructure, a recent study indicates that factors such as economic status and birth order, where a higher birth order increases the likelihood of not using skilled birth attendant services, remain key determinants of skilled birth attendant utilization [15]. These findings underscore persistent inequalities in access to maternal healthcare. Although Lumbini’s Human Development Index, reflecting health, education, and standard of living, is only slightly below the national average [16], the province’s elevated maternal mortality suggests the need for further investigation to identify underlying factors and design targeted, localized interventions.

Maternal deaths were most frequent in peri-urban and rural areas. Travel time to referral facilities is a well-recognized determinant of maternal mortality. The WHO and UNFPA recommend a maximum of two hours travel time from home to Emergency Obstetric and Neonatal Care to ensure timely intervention for women with obstetric complications [17]. Evidence from Tanzania indicates that deaths due to direct maternal causes, such as postpartum hemorrhage, increase sharply with distance, rising almost fourfold when comparing distances under 5 km to those over 35 km [18]. Similarly, in Sierra Leone, perinatal mortality increased when travel times exceeded two hours [19]. Longer travel times were often associated with transport by boat or ambulance, visits to one or two intermediary facilities before reaching the final facility, lower maternal education, and poverty [19]. In the Nepali context, the elevated maternal mortality observed in peri-urban areas likely reflects similar barriers, particularly delays in the decision to seek care and difficulties in reaching timely obstetric services. These findings emphasize the need for context-specific interventions to reduce travel-related delays and improve access to quality maternal healthcare.

Regionally, approximately half of maternal deaths occurred in the Tarai region, followed by the Hill region, and the fewest in the Mountain region. In recent years, to improve access in remote areas, the Nepalese government has repurposed military helicopters to overcome poor road infrastructure [20]. These helicopters are particularly critical for women in the most difficult-to-reach areas, such as the Mountain region, facilitating rapid transfer to fully equipped and staffed hospitals.

Socioeconomic disparities were evident: only 9.0% of deceased women belonged to wealthy households, compared with 47.7% from poor and 43.4% from middle-income households. Most maternal deaths are preventable, and social determinants, including household wealth, play a substantial indirect role in shaping disparities in maternal mortality and morbidity [4, 21]. These findings highlight the need to prioritize resources and targeted interventions for women with limited financial means. Poverty is closely associated with higher fertility and gravidity [22], factors that further increase the risk of adverse maternal outcomes and maternal mortality [21]. In addition, lower educational attainment, more common among poorer households, contributes to higher maternal mortality [21] by limiting access to essential health information and care. To help address these socioeconomic disparities in low-literacy settings, where limited maternal health knowledge is a key driver of mortality, Nepal has implemented an innovative strategy using songs to convey maternal health messages [23]. This approach has proven highly effective, with the greatest improvements observed among the most illiterate members of the community [23].

Inequalities in maternal health service use and outcomes across caste and ethnic groups persist in Nepal [24]. In our study, maternal deaths were most frequent among mountain/hill/tarai Janajati women. In contrast, previous evidence from Nepal shows that a disproportionate share of maternal deaths occurs among Terai/Madhesi and lower-caste groups, driven by barriers such as low social status, financial constraints, traditional beliefs, difficult geographic access, and service readiness gaps [25]. National analyses further demonstrate pronounced inequities in service utilization by caste, ethnicity, and wealth, with the lowest coverage of four or more antenatal care visits and skilled birth attendance consistently observed among Terai/Madhesi Dalit and the poorest households [24]. In addition, relatively higher levels of service coverage among Hill castes, as reported in national surveys, should be considered when interpreting the distribution of maternal deaths in our study [24]. Regarding religion, most deceased women were Hindu, reflecting Nepal’s overall religious composition.

Most maternal deaths occurred within six weeks postpartum, followed by fewer than one-fourth during pregnancy, and only a single case during childbirth. According to the 2021 Nepal Maternal Mortality Report, nearly one-third of postpartum deaths were attributed to obstetric hemorrhage [3], a leading cause of maternal mortality worldwide [12], while hypertensive disorders were the second most common direct cause [3]. These findings underscore the critical need for timely, high-quality, and accessible maternal care throughout pregnancy, childbirth, and the postpartum period. The adverse effects of various risk factors can be substantially mitigated through strong health systems that ensure comprehensive preconception, antenatal, intrapartum, and postpartum services [4]. Policymakers, particularly in high-burden settings such as Nepal, must also recognise that the major biomedical causes of maternal deaths are deeply interconnected with systemic and social determinants [4].

Despite the decriminalization of abortion in Nepal in 2002, unsafe abortion remains a significant contributor to maternal morbidity and mortality [26], compounded by persistent stigma, especially among unmarried women. Although verbal autopsy is a valuable tool for assessing causes of maternal death, abortion-related maternal mortality in our study is likely underestimated. This is because family members may be unaware, or reluctant to report abortions, particularly among unmarried women [27].

Priority interventions should include strengthening antenatal care with routine screening and appropriate management of conditions such as hypertension and pre-eclampsia for early detection and prevention. Ensuring the presence of skilled birth attendants competent in managing obstetric emergencies, including postpartum hemorrhage, is equally essential. Continuous training and competency maintenance for skilled providers, coupled with robust and responsive referral systems, are critical to guaranteeing timely access to emergency obstetric care and preventing avoidable maternal deaths. Additionally, addressing poverty-related barriers is crucial, and may include subsidising maternal health services, providing transportation to health facilities, implementing community-based outreach programs, and ensuring financial protection mechanisms so that low-income women can access quality care without undue economic hardship.

## Conclusion

Maternal mortality in Nepal is strongly influenced by social and geographic factors. Women from poorer households, rural and peri-urban areas, and specific provinces face disproportionately higher risks, with most deaths occurring within six weeks postpartum. Strengthening antenatal care, ensuring skilled birth attendance, and improving referral systems, while simultaneously addressing socioeconomic and geographic inequities, are essential. Targeted, context-specific interventions that guarantee equitable access to comprehensive maternal health services for all women are critical to reducing preventable maternal deaths.
